# Uric acid and glaucoma: a systematic review and meta-analysis

**DOI:** 10.3389/fmed.2023.1159316

**Published:** 2023-07-28

**Authors:** Mohammad Mohammadi, Adeleh Yarmohammadi, Amin Salehi-Abargouei, Hamidreza Ghasemirad, Mohammad Shirvani, Hamed Ghoshouni

**Affiliations:** ^1^Students' Research and Technology Committee, Shahid Sadoughi University of Medical Sciences, Yazd, Iran; ^2^NeuroTRACT Association, Student's Scientific Research Center, Tehran University of Medical Sciences, Tehran, Iran; ^3^Assil Gaur Eye Institute, Beverley Hills, CA, United States; ^4^Research Center for Food Hygiene and Safety, School of Public Health, Shahid Sadoughi University of Medical Sciences, Yazd, Iran; ^5^Yazd Cardiovascular Research Center, Non-communicable Diseases Research Institute, Shahid Sadoughi University of Medical Sciences, Yazd, Iran; ^6^Department of Nutrition, School of Public Health, Shahid Sadoughi University of Medical Sciences, Yazd, Iran; ^7^Geriatric Ophthalmology Research Center, Shahid Sadoughi University of Medical Science, Yazd, Iran; ^8^Poostchi Ophthalmology Research Center, Department of Ophthalmology, School of Medicine, Shiraz University of Medical Sciences, Shiraz, Iran

**Keywords:** glaucoma, intraocular pressure, uric acid, oxidative stress, systematic review, meta-analysis

## Abstract

**Background:**

Glaucoma, the leading cause of irreversible blindness, is a common disorder that contributes to gradual optic nerve degeneration. The beneficial impacts of uric acid (UA) have been reported in some neurodegenerative conditions such as Parkinson's disease, Alzheimer's disease, and amyotrophic lateral sclerosis. But the results of current studies about the association between serum UA level and glaucoma are conflicting. The present meta-analysis was conducted to provide a better understanding of the association between serum UA level and glaucoma.

**Methods:**

We searched the databases of PubMed, Scopus, Web of Science, and Google Scholar systematically until November 20, 2022 to identify case-control studies, comparing the serum UA concentrations of the patients with glaucoma and controls. The mean ± standard division difference was used to assess the difference in serum UA concentrations between the glaucoma patients and controls.

**Results:**

Six studies involving 1,221 glaucoma patients and 1,342 control group were included in the present meta-analysis. This meta-analysis using a random effect model indicated that the mean UA level in glaucoma patients was 0.13 (*I*^2^ = 91.92%, 95% CI = −0.42 to 0.68) higher than the controls; however, it was not statistically significant.

**Conclusions:**

Our findings provide evidence that glaucoma patients have a higher serum UA level compared to the controls, but this difference is not statistically significant. Prospective studies are needed to determine the possible association between increased UA and glaucoma pathogenesis.

**Systematic review registration:**

https://www.crd.york.ac.uk/prospero/display_record.php?ID=CRD42022364055, identifier: CRD42022364055.

## Introduction

Glaucoma is the leading cause of irreversible blindness in the world (1). The prevalence of this disorder is rising and varies globally (2, 3), and it is predicted that the number of glaucoma patients will exceed 110 million people by 2040 (4).

In the early stages, glaucoma might be asymptomatic, or patients may experience blurred or missing areas in their vision field (5). However, the late stages of the condition can result in irreversible blindness, especially if untreated (6).

Although the harmful effects of glaucoma on vision are irreversible, early diagnosis and treatment of this condition can decrease the risk of permanent blindness (7).

Due to the asymptomatic nature of glaucoma, early detection of the disease is challenging, and the number of diagnosed patients with glaucoma is lower than undiagnosed patients (8, 9).

Intraocular pressure (IOP) measurement is one of the main diagnostic tests for the diagnosis and progress monitoring of glaucoma (10). Evaluated IOP is the leading risk factor for glaucoma (11). IOP reflects the balance between the aqueous humor generation and its drainage from the eye through the trabecular meshwork and the Schlemm canal outflow pathway (12). Dysfunction of this outflow pathway elevates IOP, which results in glaucomatous optic neuropathy, but it has been shown that normal IOP also may be found in some glaucoma patients (13). This suggests that other factors may involve in the underlying mechanism of glaucoma and underline the need to prioritize research in this area to promote the clinicians' insight into the development of glaucoma.

Glaucoma has traditionally been considered an eye disease, but recent studies have linked it to central nervous system degeneration (14–16). The neurodegeneration associated with glaucoma contributes to gradual optic nerve degeneration with progressive retinal ganglion cell (RGC) loss, which is the main cause of progressive vision loss (17, 18).

The underlying pathogenesis of glaucomatous optic neuropathy is still unknown. It has been suggested that glaucoma destroys neurons through neuroinflammation and oxidative stress (18). Antioxidants can be protective against glaucoma through different mechanisms such as IOP reduction, promoting vascular health, and prevention of RGC loss (19).

Uric acid (UA) is a purine metabolite that detects intracellularly and in all body fluids (20) and that shown to have both pro-oxidant and antioxidant features *in-vitro* by production and scavenging of reactive oxygen species (21, 22). The beneficial impacts of UA have been shown in other neurodegenerative conditions, such as Parkinson's disease (23), Huntington's disease (24), Alzheimer's disease (25), and amyotrophic lateral sclerosis (26). However, the role of UA in the underlying mechanism of glaucoma is still unclear.

By exploring the association between uric acid and glaucoma, we can identify potential abnormal metabolic processes in glaucoma patients, thereby considering UA as a biomarker. Data from several case-control studies suggest a significant inverse association between serum concentrations of UA and glaucoma risk (27–29). However, this was not confirmed in all studies, and some studies even reported a significant association between high serum UA concentrations and the risk of glaucoma (30–32). Hence, the current meta-analysis aimed to evaluate the relationship between serum UA concentration and glaucoma in case-control studies.

## Method

The present study was conducted according to the Preferred Reporting Items for Systematic Reviews and Meta-Analyses (PRISMA) (33). The protocol has been registered in the PROSPERO database (registration number: CRD42022364055).

### Eligibility criteria

The present systematic review focused on case-control studies that reported the serum UA concentrations in patients with glaucoma and compared them to controls. The investigations should be conducted *in vivo* in humans, and the subjects could be controls or glaucoma patients.

We included only studies in the English language. We excluded conference meetings and abstracts that were not published in peer-reviewed journals. If original data or exact numbers were unavailable in both groups of patients with glaucoma and controls, they were not included in our quantitative analysis.

### Search strategy and literature screening

To identify studies to be included in this review, a systematic search was performed via PubMed, Scopus, Google Scholar, and Web Of Science databases from inception through November 20, 2022. We used the following search strategy: (“uric acid”[Mesh]) AND (“glaucoma”[Mesh] OR “Intraocular Pressure”[Mesh] OR “Ocular Hypertension” [Mesh]). Moreover, the reference lists of studies obtained in the initial search were manually searched for more relevant articles.

### Study selection

After removing duplicate records, two reviewers (MM and HRG) independently analyzed all titles and abstracts obtained from the searches to identify relevant papers.

The full text of studies that appeared to meet the inclusion criteria were obtained and independently analyzed by two reviewers (MM and HG). The authors resolved the disagreement through a discussion with a third author (AS). Eventually, studies that did not meet the inclusion criteria were discarded.

### Risk of bias assessment

We applied the Newcastle-Ottawa Quality Assessment Scale (NOS) for case-control studies to evaluate the methodological quality of the studies (34). The NOS contains a star system in which a study is assessed on three domains (Supplementary Table S1); representativeness of study group selection (four items), comparability of groups (two items), and ascertainment of the exposure (three items). Studies scored one star for each area addressed, with a maximum score of 9, of which 7–9, 4–6, 0–3, and scores considered high, fair, low, and quality, respectively. Disagreements were resolved by discussion, and a third author arbitrated unresolved discrepancies.

### Data extraction

Using standardized data extraction forms, two reviewers (MM and AS) extracted data independently. In cases of disagreement between the reviewers, a third reviewer (HG) was consulted. The following data were extracted from selected studies: authors' name, publication year, country of study, glaucoma type, number of subjects, age range of subjects, definition, and the mean and standard deviation of serum UA levels. Data were extracted separately for each entity groups (glaucoma patients or controls).

### Statistics

The mean difference (MD) and its corresponding standard error (SE) were calculated by using the mean values and their standard deviations reported/calculated for case and control groups. Then MDs extracted from each study were used as effect size for meta-analysis. The meta-analyses were performed using DerSimonian-Laird random-effects model, which takes the between-study heterogeneity into account. Stata software, version 17.0 (Stata Corp, College Station, TX), was used to analyze the data. Both the Q statistic and *I*^2^ statistic measures were used for the evaluation of heterogeneity between studies. *P*-values < 0.05 for Cochran's Q test and an *I*^2^ higher than 25% will be considered as significant heterogeneity (35).

*P*-values < 0.05 were considered statistically significant. To conduct a sensitivity analysis, each article was removed from the final analysis. Begg's funnel plots and Egger's and Begg's asymmetry tests were used to assess the presence of publication bias (36).

## Result

### Study selection

**Figure 1:**
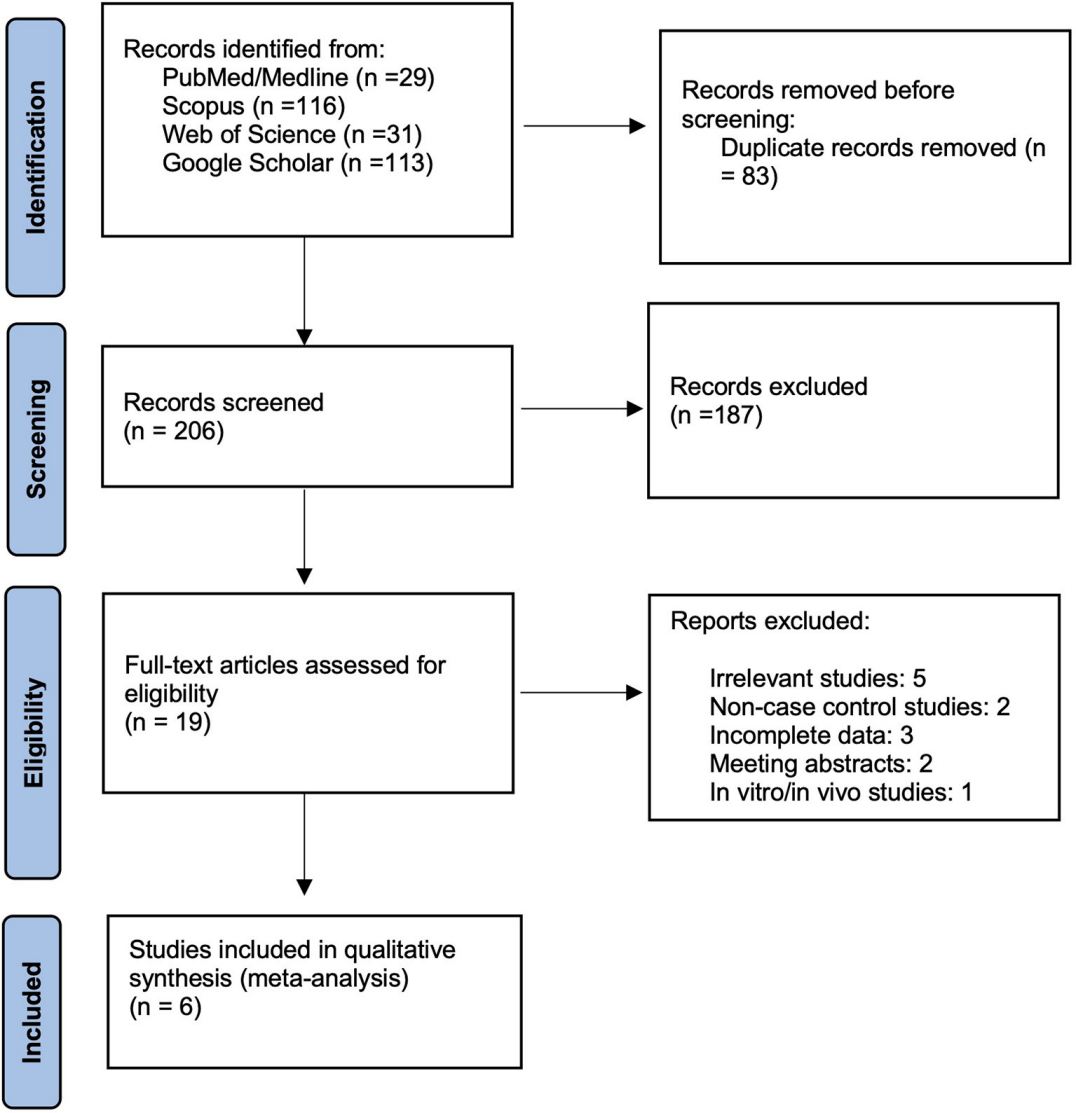
PRISMA flow diagram of included studies.

After the systematic search in databases, 289 records were retrieved. By removing 83 duplicate records, 206 were screened, and finally, the six studies met the inclusion criteria and were included in this meta-analysis. The Prisma flowchart shows this process in detail in Figure 1.

### Study characteristics

Among six studies evaluating the level of UA in serum, 1,221 glaucoma patients were compared to the 1,342 control group. One study recruited patients with normal-tension glaucoma (NTG), another study with primary angle closure glaucoma (PACG), while the patients in the other four studies all were primary open-angle glaucoma (POAG). The largest sample size between these studies included 886 primary angle closure glaucoma patients, with 994 participants as a control group. In comparison, the smallest contained 23 primary open-angle glaucoma patients with 15 participants as a control group. These studies were performed in China (*n* = 2), Tunis (*n* = 1), Greece (*n* = 1), Italy (*n* = 1), and Japan (*n* = 1).

The mean UA level across all glaucoma patients ranged between 4.00 ± 0.66 mg/dl In Serra et al. to 6.2 ± 1.9 mg/dl in Elisaf et al. The characteristics of each included study are shown in Table 1.

**Table 1:** Characteristics of the six included studies in the present meta-analysis.

**References**	**Country**	**Glaucoma type**	**Patients**				**Controls**			
			**Number (M/F)**	**Age range**	**Definition**	**Serum UA level (mg/dl)**	**Number (M/F)**	**Age range**	**Definition**	**Serum UA level (mg/dl)**
Bouchemi et al. (32)	Tunis	POAG	100 (46/54)	68.33 ± 1.44	IOP > 21 mm Hg + open-angle glaucoma + visual field loss + glaucomatous optic nerve head alternations	5.44 ± 4.30	114 (52/62)	70.18 ± 1.14	Normal IOP + senile cataract + not received topical drugs	4.55 ± 2.77
Elisaf et al. (30)	Greece	POAG	49 (34/15)	65 ± 9	Visual field defect + optic disk damage on the open-angle of anterior chamber + deep anterior chamber	6.2 ± 1.9	72 (52/20)	63 ± 8	Normal IOP	5.0 ± 1.2
Li et al. (29)	China	POAG	163 (108/55)	49.99 ± 17.24	Inpatients scheduled for glaucoma surgery + age ≥ 18 years + open anterior chamber angle + visual field with characteristic glaucomatous damage consistent with nerve fiber layer loss	5.4 ± 1.41	103 (77/26)	51.42 ± 16.14	IOP < 21 mm Hg + age ≥ 18 years + open anterior chamber angle + VCDR ≤ 0.5 + no family/personal history of glaucoma + no prior eye surgery + no systemic disease	6.09 ± 0.89
Li et al. (28)	China	PACG	886 (302/584)	63.17 ± 10.65	IOP > 21 mm Hg + narrow eye angles + at least 180 of angle-closure obliterating the pigmented part of the trabecular meshwork + too extensive degree of peripheral anterior synechiae to be managed by laser peripheral iridotomy	4.81 ± 1.38	994 (370/624)	63.26 ± 10.12	N/d	4.96 ± 1.43
Serra et al. (27)	Italy	POAG	23 (10/13)	68.68 ± 7.54	IOP > 21 mm Hg+ visual field defect + optic disk damage + open iridocorneal angle + deep anterior chamber	4.00 ± 0.66	15 (5/10)	65 ± 4.56	BCVA ^*d*^≥ 0.0 logMAR + IOP < 21 mm Hg + no glaucomatous optic nerve head alterations + no family history of optic nerve head diseases + no cause of hyperuricemia	4.95 ± 0.86
Yuki et al. (31)	Japan	NTG	47 (18/29)	59.5 ± 10.2	Non-occludable and open anterior chamber angles + glaucomatous optic disc cupping + visual field defect	5.8 ± 1.5	44 (16/28)	62.7 ± 14.8	N/d	4.9 ± 1.4

M/F, male/female; VCDR, vertical cup-to-disc ratio; N/d, not defined; BCVA, best corrected visual acuity.

For quality assessment of included studies, the Newcastle-Ottawa Scale score was used. Almost all studies have good-quality scores, and Supplementary Table S1 shows these scores in detail.

### Results of syntheses

**Figure 2:**
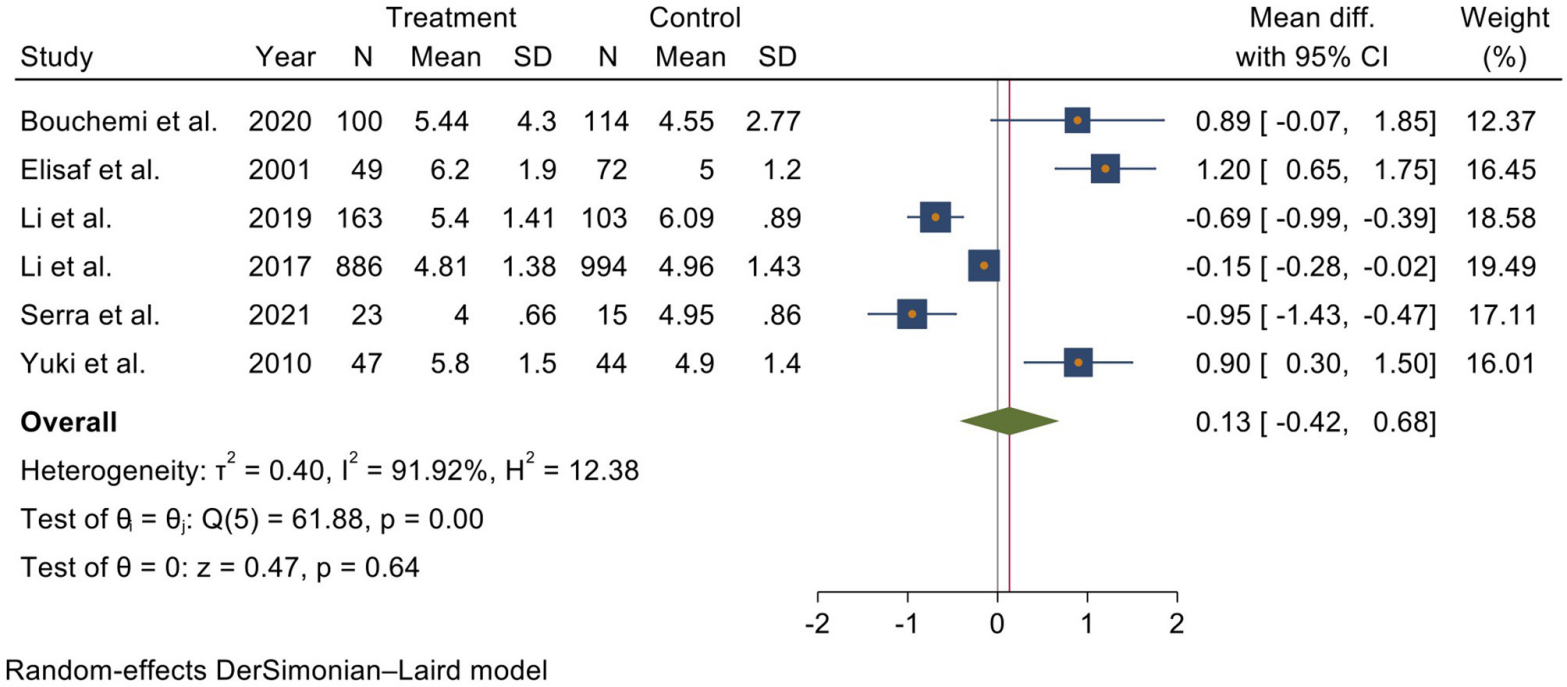
Forest plot of meta-analysis for serum uric acid level in glaucoma patients and control groups.

The pooled analysis included all six studies, showing that serum UA level was higher in glaucoma patients than in other patients without glaucoma. In detail, A meta-analysis using a random effect model indicates that the mean UA level in glaucoma patients was 0.13 (*I*^2^ = 91.92%, 95% CI = −0.42 to 0.68) higher than the controls; however, it was not statistically significant (Figure 2).

Each article was removed from the analysis to perform a sensitivity analysis, and no significant effect of a single study was found. The funnel plot and Begg and Egger test showed no evidence of publication bias (*P* > 0.05) (Supplementary Figure S1).

## Discussion

So far, the association between serum UA concentrations and glaucoma is under debate. To the best of our knowledge, this is the first meta-analysis to examine the relationship between serum UA level and glaucoma.

In the present meta-analysis of 1,221 glaucoma patients compared to 1,342 controls included in 6 case-control studies, serum UA concentration in patients with glaucoma was higher than in the controls, but this association was not statistically significant.

Three out of six case-control studies (27–29) within this meta-analysis found a significant inverse association. In comparison, three other studies (30–32) have reported a positive association between high UA levels and glaucoma (Table 1). These various results may be reflected by the heterogeneity found in the present meta-analysis. The inclusion of various glaucoma types in this meta-analysis and the differences in disease etiology could contribute to this heterogeneity (Table 1).

Some authors have recently found decreased total antioxidant capacity levels in the blood and aqueous humor samples of glaucoma patients (37, 38). Oxidative stress is also suggested to play a role in the physiologic changes in aqueous humor outflow, leading to increased IOP and RGC degeneration in glaucoma (39, 40).

UA is one of the main antioxidants of plasma (21, 41). Wayner et al. (42) reported that urate contributes up to 65% of the overall antioxidant capacity of the plasma. Meanwhile, experimental animal studies and human clinical trials have suggested that higher serum UA concentrations can prevent neuronal degeneration (43, 44).

Li et al. investigated the association between the progression of recently diagnosed PACG and pretreatment UA levels of serum. In this prospective observational study, there was a correlation between a lower baseline serum UA concentration and a higher risk of PACG progression. These findings suggested that higher serum UA values may protect against PACG and suppress the disease progression (45).

On the other hand, some studies have suggested that systemic inflammation is related to glaucomatous damage (46). A recent study by Astafurov et al. (46) showed that glaucoma patients had greater bacterial oral counts in compression to controls and low-dose lipopolysaccharide administration in glaucoma animal models led to neuronal loss and axonal degeneration. In addition, recent studies have reported a significant association between *Heliobacter pylori* infection and glaucoma (47, 48).

As mentioned above, it seems that glaucoma patients are in a low antioxidative and high oxidative state in the body. UA may be consumed in glaucoma by preferentially reacting with oxidizing agents in the body. These findings are consistent with former studies reporting that subjects with higher UA levels have a decreased risk of glaucoma (27–29) and that the level of UA was negatively related to the glaucoma severity (28, 29).

However, another previous study compared the serum UA levels between pseudoexfoliation patients (the leading cause of secondary glaucoma) and controls and reported that serum UA levels of subjects with and without pseudoexfoliation were similar (49).

The other three studies included in our meta-analysis (30–32) suggested higher serum UA concentrations were found in glaucoma patients in comparison with controls.

Regarding this subject interestingly, elevated levels of UA have been reported in the aqueous humor of some patients with glaucoma (50). Additionally, it has been suggested that oxidative stress can accelerate the apoptosis of trabecular meshwork cells and extracellular matrix accumulation in the trabecular meshwork, leading to increased resistance of the aqueous humor outflow pathway and an increase in IOP (51). It is possible that elevated serum UA may reduce the outflow facility of aqueous humor by impairing the trabecular meshwork physiology, ultimately leading to an increase in IOP and glaucomatous optic neuropathy.

Nevertheless, IOP elevation is insufficient to explain the underlying pathophysiology of glaucoma (52). Therefore, other involving risk factors, particularly the impairment of the vasculature supplying the optic nerve and the tissues around it, have also been suggested (53).

According to the growing body of clinical and experimental research, UA-induced inflammatory response and oxidative stress contribute to microvascular impairments (54, 55). Some *in vitro* and *in vivo* findings suggested that UA may contribute to endothelial dysfunction by causing antiproliferative impacts on the endothelium (56, 57), which has been shown to have an important association with open-angle glaucoma (58).

Moreover, it has been reported that an elevated serum UA and its fluctuations were independently related to impaired choroidal and retinal microcirculation (59).

In a recent study, Yang et al. reported that higher serum UA concentrations were noticeably associated with decreased retinal capillary plexus vessel density. These results may support the damaging impact of high serum UA concentrations on the retinal microvasculature and suggest the necessity of regulating serum UA to prevent microvascular alteration (60). In addition, interestingly, it has been reported that a history of chronic renal disease is significantly associated with the higher risk of development of subsequent glaucoma (61).

Serum UA is known as a potential risk factor for the development and progression of chronic renal disease. It has been reported that elevated serum UA levels can cause an increase in glomerular blood pressure leading to renal diseases. Additionally, pilot studies have suggested that lowering serum UA therapies may slow the progression of chronic renal disease (62). In this regard, it is suggested that both the choroid plexus in the human eye and the renal glomerulus have extensive vascular networks with similar structures (63). The underlying mechanism of glaucoma development may be similar to the chronic renal disease. According to these findings, it is possible that higher levels of UA may be contributing to glaucomatous optic neuropathy.

With all these interpretations, this study was a meta-analysis of the case-control studies, and we cannot consider a precise causal role for UA in the pathogenesis of glaucoma.

Due to the limited number of primary studies available, we were unable to perform a separate analysis for UA levels in each glaucoma subtype. Therefore, it is important to interpret the results cautiously, considering the potential variations in UA levels among different glaucoma types.

In addition, systemic diseases and some medications administration may affect the serum UA level (64–66). Except for Bouchemi et al. (32), all the studies analyzed in this meta-analysis excluded individuals with systemic diseases or those taking medications that could affect serum UA levels. Bouchemi et al. (32) did not clearly state the criteria for excluding patients with systemic diseases or those using medications that could impact serum UA levels.

The observational nature of the included studies does not allow us to determine whether UA-lowering interventions can influence the development of glaucoma. Further randomized clinical trials are required to assess whether the UA-lowering medications may be beneficial in managing glaucoma.

The primary objective of our study was to compare serum UA levels between patients with glaucoma and the control group. We did not analyze and compare the concentration of UA levels in the vitreous and/or aqueous humor. Prior studies have shown that UA levels in aqueous humor of patients with glaucoma were higher than controls (32, 50). The exact mechanism by which UA is transferred into the aqueous humor remains unclear. However, several urate transporters that are involved in UA homeostasis, such as the ATP-binding cassette transporters, organic anion transporters, and solute carrier transporters have been identified in the retina and/or ciliary body of human eyes (67–70). These transporters may be involved in regulating of UA levels in human eyes. Future studies should consider comparing the UA levels in the vitreous and/or aqueous humor to gain a more comprehensive insight into its role in the development of glaucoma.

Furthermore, the study population of included studies in our meta-analysis was limited, and it is necessary to conduct more extensive prospective cohort studies to determine the potential link between serum UA levels and glaucoma.

## Conclusion

This meta-analysis summarized a large body of evidence from case-control studies on the association between serum UA level and glaucoma. These findings provided evidence that serum UA concentrations are higher in glaucoma patients in comparison with controls, but this association is not statistically significant. However, prospective studies are needed to confirm the exact effect of serum UA concentrations on the risk of glaucoma.

## Data availability statement

The original contributions presented in the study are included in the article/Supplementary material, further inquiries can be directed to the corresponding author.

## Author contributions

MM and HGho designed the study. HGha, MM, and AY performed a systematic search and extracted the data. AS-A and HGho conducted statistical analysis. MM, HGho, and MS drafted the paper. All authors read, revised the manuscript, contributed to the article, and approved the submitted version.
