# Supplementary material for: Uric acid and glaucoma: a systematic review and meta-analysis
Source: Front Med (Lausanne). 2023 Jul 28;10:1159316. doi: 10.3389/fmed.2023.1159316 (PMC10422028; doi:10.3389/fmed.2023.1159316)
Supplement: Supplementary file 1 [file Data_Sheet_1.docx]

Table 1S. Quality assessment of included studies based on the Newcastle-Ottawa Scale score

| First author, year | Selection | | | | | Comparability of cases and controls on the basis of the design or analysis | Exposure | | | Total stars |
| --- | --- | --- | --- | --- | --- | --- | --- | --- | --- | --- |
|  | Is the case definition adequate | Representativeness of the cases | Selection of controls | Definition of controls | |  | Ascertainment of exposure | Same method of ascertainment for cases and controls | None-response rate |  |
| Bouchemi, 2020 | * | * | - | * | | - | * | * | * | 6 |
| Elisaf, 2001 | * | * | - | * | | * | * | * | * | 7 |
| Li, 2018 | * | * | * | * | | * | * | * | * | 8 |
| Li, 2016 | * | * | * | * | | * | * | * | * | 8 |
| Serra, 2020 | * | * | * | | * | - | * | * | * | 7 |
| Yuki, 2009 | * | * | * | | * | - | * | * | * | 7 |

* Each asterisk represents if individual criterion within the subsection was fulfilled.


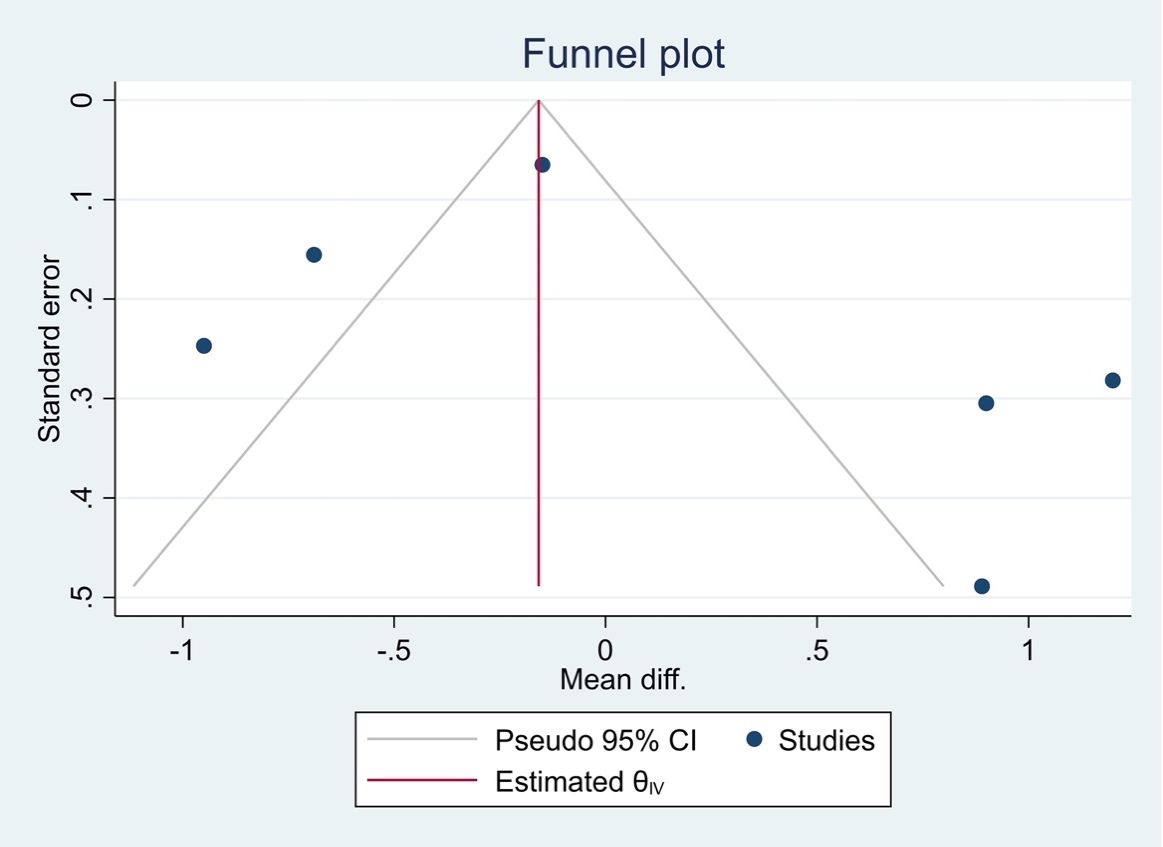


Fig 1S. Funnel plot of the publication bias.
